# Significance of Neoadjuvant S‐1‐Based Chemotherapy for Older Patients With Locally Advanced Gastric Cancer

**DOI:** 10.1002/ags3.70049

**Published:** 2025-05-31

**Authors:** Kota Kawabata, Takuro Saito, Yukinori Kurokawa, Kazuyoshi Yamamoto, Tsuyoshi Takahashi, Koji Tanaka, Tomoki Makino, Kiyokazu Nakajima, Hidetoshi Eguchi, Yuichiro Doki

**Affiliations:** ^1^ Department of Gastroenterological Surgery Osaka University Graduate School of Medicine Suita Japan

**Keywords:** gastric cancer, neoadjuvant chemotherapy, older patients, preoperative DOS, preoperative SOX

## Abstract

**Background:**

Neoadjuvant chemotherapy (NAC) has been extensively developed for locally advanced gastric cancer (GC). In Asia, S‐1‐based regimens, such as docetaxel, oxaliplatin, and S‐1 (DOS) and S‐1 and oxaliplatin (SOX), are expected to become the standard of care. However, the data on the significance of NAC for older patients with advanced GC remains scarce. Therefore, this study aimed to evaluate the feasibility and efficacy of NAC in older patients.

**Methods:**

We retrospectively analyzed the data from patients with cStage II–III locally advanced GC who underwent radical surgery at our institution between 2015 and 2021. This study included three groups: 56 patients with NAC and age < 75 years (NAC‐Young group), 20 with NAC and age ≥ 75 years (NAC‐Older group), and 46 without NAC and age ≥ 75 years (OP‐Older group). Patient backgrounds, adverse events of NAC, and prognoses were compared among the groups.

**Results:**

Compared with the NAC‐Young group, the NAC‐Older group was more likely to receive the SOX regimen and reduced initial doses, but there was no significant difference in the incidence of adverse events of NAC and prognosis. Compared to the OP‐Older group, overall survival and cancer‐specific survival tended to be better in the NAC‐Older group at cStage III. Moreover, for patients with cStage III and ECOG‐PS 0, cancer‐specific survival was significantly better in the NAC‐Older group compared to the OP‐Older group (*p* = 0.030).

**Conclusions:**

NAC with S‐1‐based regimens is a feasible and effective treatment option for older patients with GC with advanced‐stage disease and good overall condition.

## Abstract 2

This study revealed that neoadjuvant S‐1‐based chemotherapy can be safely and effectively administered to older patients with locally advanced gastric cancer by adjusting chemotherapy regimens and doses.

## 1 Introduction

Gastric cancer (GC) is the fifth most common cancer globally [1, 2]. The standard treatment for curatively resectable locally advanced GC is surgery plus perioperative chemotherapy, and various treatment strategies have been developed to improve prognosis [3, 4]. In Europe, perioperative chemotherapy with fluorouracil, leucovorin, oxaliplatin, and docetaxel (FLOT) regimen is the present standard therapy [5], whereas in Asia, upfront surgery plus adjuvant chemotherapy is the standard therapy; however, neoadjuvant chemotherapy (NAC) has been developed vigorously [6]. The Korean phase‐III PRODIGY trial demonstrated an improved prognosis of NAC with docetaxel, oxaliplatin, and S‐1 (DOS) regimen in locally advanced GC [7, 8]. The Chinese phase‐III RESOLVE trial also demonstrated the efficacy of perioperative chemotherapy with S‐1 and oxaliplatin (SOX) regimen for locally advanced GC [9]. In addition, in Japan, several clinical trials are currently underway to investigate the benefit of NAC‐DOS or NAC‐SOX for GC and esophagogastric junction cancer (EGC) [10, 11]. Based on the results of the recent phase‐III PRODIGY and phase‐III RESOLVE trials in Asia, preoperative DOS and SOX therapies are expected to become the standard of care for advanced GC, replacing the treatment strategy with upfront surgery [7, 8, 9].

The proportion of older patients with advanced GC is increasing with the growing older population in Japan, where it is assumed that the proportion of older patients aged ≥ 75 years will exceed 60% of all patients with GC in 10 years [12]. However, in most clinical trials, such as the PRODIGY trial, the inclusion criteria are often restricted to patients aged 75–80 years or younger with a good performance status. Consequently, in practice, these trials predominantly include younger patients with good performance status. Thus, the feasibility and efficacy of NAC for older patients with GC and EGC are unknown.

In our hospital, we have treated patients with advanced GC and EGC with NAC‐DOS or NAC‐SOX, including older patients. Therefore, this study aimed to retrospectively evaluate the feasibility and efficacy of NAC‐DOS or NAC‐SOX in older patients with locally advanced GC and EGC.

## 2 Materials and Methods

### 2.1 Patient Population

**FIGURE 1:**
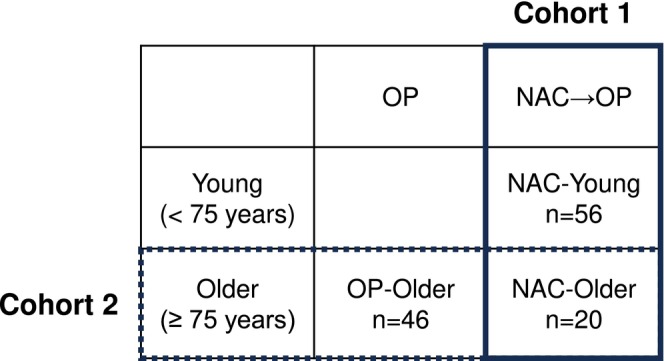
Schema of the two cohorts. Cohort 1 (solid line) includes 76 patients who underwent surgery after neoadjuvant chemotherapy and are divided into two groups: NAC‐Young (< 75 years, *n* = 56) and NAC‐Older groups (75 years ≤, *n* = 20). Cohort 2 (dotted line) includes 66 older patients aged ≥ 75 years and is divided into two groups: OP‐Older (≥ 75 years, *n* = 46) and NAC‐Older groups (≥ 75 years, *n* = 20).

This retrospective cohort study included patients with cStage II/III locally advanced GC or EGC who underwent radical surgery at Osaka University Hospital between June 2015 and June 2021. Patients with concurrent carcinoma in situ were excluded. All patients had histologically confirmed primary GC or EGC and were classified as having cStage II/III disease based on endoscopic examination and contrast‐enhanced computed tomography (CT) scanning before treatment. Tumor staging was based on the 8th Edition of the Union for International Cancer Control TNM Classification of Malignant Tumors. The primary targets of NAC at our hospital were cStage II–III EGC and cStage III GC; however, the feasibility, regimen, and dosage depended on the patient's condition. In older patients (aged ≥ 75 years), NAC was selectively indicated for those with good overall condition, taking into account for ECOG‐PS and the presence or absence of comorbidities, as determined by the attending physician. Among patients with NAC, 4 patients who did not undergo gastrectomy due to disease progression after NAC were excluded, 3 under 75 years old and 1 over 75 years old. A total of 122 patients were included in this study, and we examined using two cohorts (Figure 1). To evaluate the feasibility of NAC in older patients, 76 patients with NAC‐DOS or NAC‐SOX were enrolled in cohort 1, which included the NAC‐Young group (< 75 years, *n* = 56) and NAC‐Older group (≥ 75 years, *n* = 20). Then, to evaluate the efficacy of NAC in older patients, 66 patients of age ≥ 75 years were enrolled in cohort 2, which included the OP‐Older group (*n* = 46) and NAC‐Older group (*n* = 20). Various factors, including patient characteristics and perioperative and postoperative factors, were compared in these cohorts. The Human Ethics Review Committee of Osaka University Graduate School of Medicine approved this retrospective study (Approval ID: 21440). All patients provided written informed consent for clinical data use before treatment, as required by the Institutional Review Board of the Osaka University of Medicine.

### 2.2 NAC and Preoperative Examinations

Patients received the DOS regimen, but the SOX regimen was considered according to the patient's condition accounting for age and comorbidities. DOS regimen consisted of docetaxel (40 mg/m^2^) and oxaliplatin (100 mg/m^2^) intravenously on day 1, with oral S‐1 twice a day at a dose based on body surface area (< 1.25 m^2^, 40 mg; ≥ 1.25 to < 1.5 m^2^, 50 mg; ≥ 1.5 m^2^, 60 mg) from days 1 to 14 for three 3‐week cycles. SOX regimen consisted of oxaliplatin (100 mg/m^2^) intravenously on day 1, with oral S‐1 twice a day at a dose based on body surface area (< 1.25 m^2^, 40 mg; ≥ 1.25 to < 1.5 m^2^, 50 mg; ≥ 1.5 m^2^, 60 mg) from days 1 to 14 for three 3‐week cycles. During each cycle, S‐1 was discontinued if patients had a neutrophil count < 500/mm^3^, platelet count < 50 × 10^3^/mm^3^, aspartate aminotransferase or alanine transaminase > 100 IU/L, total bilirubin > 3.0 mg/dL, creatinine > 1.5 mg/dL, or non‐hematological toxicity of grade 2 or higher. Toxicities and adverse events were evaluated throughout each course and graded according to the Common Terminology Criteria for Adverse Events, version 5.0. Three cycles of chemotherapy were planned, followed by radical surgery. CT scans that included the chest and the whole abdomen were carried out after cycles 1 and 3 to evaluate the tumor response. If tumor progression was confirmed after cycle 1, NAC was discontinued and surgical resection was planned. An endoscopic examination was carried out after cycle 3. Surgical resection was performed within 2–4 weeks after completion of NAC.

### 2.3 Surgical Treatment and Adjuvant Chemotherapy

For GC, the surgical procedures of distal, proximal, or total gastrectomy with D2 lymph node dissection were decided according to tumor location following the Japanese GC treatment guidelines. Regarding EGC, subtotal esophagectomy plus proximal gastrectomy was performed for patients with esophageal involvement > 3 cm or clinical node‐positive disease in the upper or middle mediastinal field. However, lower esophagectomy plus proximal or total gastrectomy was performed for the other patients. The severity of postoperative complications was evaluated according to the Clavien–Dindo classification system [13, 14]. All resected specimens were examined by pathologists, and tumor regression grade after chemotherapy was quantified according to the Japanese classification of GC regression criteria [15]. Regarding adjuvant chemotherapy, docetaxel and S‐1 (DS) or S‐1 monotherapy were considered according to the pathological stages, but that depended on the patient's condition and physician's choice.

### 2.4 Statistical Analysis

Data were analyzed using the JMP17 software (SAS Institute Inc., Cary, NC, USA). Continuous data were presented as medians and ranges. Group differences were analyzed using the Chi‐square and Mann–Whitney *U* tests. Kaplan–Meier survival curves and log‐rank analysis demonstrated survival differences between the curves. Overall survival (OS) was defined as the time from the date of treatment initiation to the date of death from any cause. Cancer‐specific survival (CSS) was defined as the time from the date of treatment initiation to the date of cause‐specific death. The differences in OS and CSS between the groups were tested. Subgroups were predefined according to the following baseline patient characteristics: sex, Eastern Cooperative Oncology Group performance status (ECOG‐PS), American Society of Anesthesiologists physical status (ASA‐PS), body mass index (BMI), histological type, tumor location, clinical T and N categories, and clinical stage. Hazard ratios and 95% confidence intervals (CIs) were calculated using a Cox proportional hazards model. Statistical significance was set at *p* < 0.05.

## 3 Results

### 3.1 Comparison Between NAC‐Older and NAC‐Young Groups (Cohort 1)

#### 3.1.1 Patient Characteristics

We first investigated the results of NAC between NAC‐Older and NAC‐Young groups. Table 1 summarizes the patient background characteristics. There were 20 and 56 patients in the NAC‐Older and NAC‐Young groups, respectively. The median ages were 76.5 (75–85) years in NAC‐Older and 68 (35–74) years in NAC‐Young groups. No significant differences in sex, ECOG‐PS, ASA‐PS, BMI, histological type, and tumor location were observed and no patient was ECOG‐PS 2–3. Regarding clinical stages, the NAC‐Older group tended to have fewer cT4 and cStage III diseases.

**TABLE 1:** Comparison of patient characteristics.

	NAC‐Older	NAC‐Young	*p*	OP‐Older	*p*
	*n* = 20	*n* = 56		*n* = 46	
Age, years					
Median (range)	76.5 (75–85)	68 (35–74)	< 0.001	81 (75–90)	< 0.001
Sex, *n* (%)					
Male	15 (75.0)	37 (66.0)	0.45	30 (65.2)	0.42
Female	5 (25.0)	19 (33.9)		16 (34.7)	
ECOG‐PS, *n* (%)					
0	15 (75.0)	49 (87.5)	0.20	24 (52.1)	0.17
1	5 (25.0)	7 (12.5)		18 (39.1)	
2	0 (0)	0 (0)		3 (6.5)	
3	0 (0)	0 (0)		1 (2.1)	
ASA‐PS, *n* (%)					
1	6 (30.0)	23 (41.0)	0.66	9 (19.5)	0.53
2	13 (65.0)	31 (55.3)		32 (69.5)	
3	1 (5.0)	2 (3.5)		5 (10.8)	
BMI, kg/m^2^					
Median (range)	22.1 (15.3–28.4)	21.6 (14.3–28.9)	0.58	20.8 (14.3–25.6)	0.19
Histological type, *n* (%)					
Differentiated	11 (55.0)	23 (41.0)	0.28	23 (50.0)	0.70
Undifferentiated	9 (45.0)	33 (58.9)		23 (50.0)	
Tumor location, *n* (%)					
Esophagogastric junction	6 (30.0)	18 (32.1)	0.97	3 (6.5)	0.082
Upper	4 (20.0)	9 (16.0)		9 (19.5)	
Middle	4 (20.0)	13 (23.2)		10 (21.7)	
Lower	6 (30.0)	16 (28.5)		24 (52.1)	
cT category, *n* (%)					
2	0 (0)	0 (0)	0.018	2 (4.3)	0.23
3	7 (35.0)	6 (10.7)		9 (19.5)	
4a	13 (65.0)	45 (80.3)		33 (71.7)	
4b	0 (0)	5 (8.9)		2 (4.3)	
cN category, *n* (%)					
0	4 (20.0)	4 (7.1)	0.11	11 (23.9)	0.69
1	5 (25.0)	30 (53.5)		16 (34.7)	
2	9 (45.0)	19 (33.9)		17 (36.9)	
3	2 (10.0)	3 (5.3)		2 (4.3)	
cStage, *n* (%)					
II	5 (25.0)	5 (8.9)	0.083	17 (36.9)	0.33
III	15 (75.0)	51 (91.0)		29 (63.0)	

Abbreviations: ASA‐PS, American Society of Anesthesiologists physical status; BMI, body mass index; ECOG‐PS, Eastern Cooperative Oncology Group performance status; EGJ, esophagogastric junction.

#### 3.1.2 Details of NAC and the Incidence of Adverse Events

Table 2 summarizes the details of NAC findings. The NAC‐Older group was more likely to receive NAC‐SOX (*p* = 0.053) and a reduced initial dose of chemotherapy (*p* = 0.021). However, the percentage of dose reduction in the middle course of chemotherapy was not different between the two groups. There was no significant difference in the incidence of grade 3 or more hematological and non‐hematological toxicities.

**TABLE 2:** Comparison of neoadjuvant chemotherapy findings.

	NAC‐Older	NAC‐Young	*p*
	*n* = 20	*n* = 56	
Regimen, *n* (%)			
DOS	14 (70.0)	50 (89.2)	0.053
SOX	6 (30.0)	6 (10.7)	
Number of cycles, *n* (%)			
3	11 (55.0)	45 (80.3)	0.094
2	5 (25.0)	7 (12.5)	
1	4 (20.0)	4 (7.1)	
Reduction of initial dose, *n* (%)			
Yes	5 (25.0)	3 (5.3)	0.021
No	15 (75.0)	53 (94.6)	
Dose reduction in the middle, *n* (%)			
Yes	9 (45.0)	36 (64.2)	0.13
No	11 (55.0)	20 (35.7)	
Adverse events^a^ grade 3 ≤, *n* (%) hematological toxicity			
Yes	16 (80.0)	43 (76.7)	0.76
No	4 (20.0)	13 (23.2)	
Adverse events^a^ grade 3 ≤, *n* (%) non‐hematological toxicity			
Yes	10 (50.0)	24 (42.8)	0.58
No	10 (50.0)	32 (57.1)	

Abbreviations: DOS, docetaxel, oxaliplatin, and S‐1; SOX, S‐1 and oxaliplatin.

^a^ Common Terminology Criteria for Adverse Events, version 5.0—JCOG.

#### 3.1.3 Perioperative and Pathological Findings

Table 3 summarizes the details of perioperative and pathological findings. There were no significant differences in the surgical findings between the two groups. R0 resection was achieved in 100% of the NAC‐Older group and 98.2% of the NAC‐Young group. The incidence of postoperative complications (Clavien–Dindo ≥ grade II) was not different between the two groups (*p* = 0.73). The NAC‐Older group tended to have lower ypT diseases than the NAC‐Young group, and tumor regression grade was not different between the two groups. Grade 3 pathological complete response (pCR) and major pathological response (≥ grade 2) was observed in 20.0% and 35.0% of the NAC‐older group, respectively. Adjuvant chemotherapy was administered less frequently in the NAC‐Older group (*p* = 0.006).

**TABLE 3:** Comparison of perioperative and pathological findings.

	NAC‐Older	NAC‐Young	*p*	OP‐Older	*p*
	*n* = 20	*n* = 56		*n* = 46	
Surgical approach, *n* (%)					
Robotic	0 (0)	1 (1.7)	0.68	5 (10.8)	0.10
Laparoscopic	14 (70.0)	41 (73.2)		33 (71.7)	
Open	6 (30.0)	14 (25.0)		8 (17.3)	
Surgical procedure, *n* (%)					
Total gastrectomy	7 (35.0)	17 (30.3)	0.66	9 (19.5)	< 0.001
Distal gastrectomy	7 (35.0)	20 (35.7)		31 (67.3)	
Proximal gastrectomy	1 (5.0)	8 (14.2)		6 (13.0)	
Subtotal esophagectomy	5 (25.0)	11 (19.6)		0 (0)	
Reconstruction					
Billroth‐I	1 (5.0)	6 (10.7)	0.84	4 (8.7)	0.024
Billroth‐II	1 (5.0)	3 (5.3)		16 (34.7)	
Roux‐en‐Y	12 (60.0)	29 (51.7)		20 (43.4)	
Other	6 (30.0)	18 (32.1)		6 (13.0)	
Operative time (min)					
Median (range)	326 (224–630)	387 (197–704)	0.073	291.5 (178–610)	0.47
Blood loss (mL)					
Median (range)	90 (20–1060)	130 (0–1620)	0.77	20 (0–1950)	0.001
Residual tumor, *n* (%)					
R0	20 (100)	55 (98.2)	0.43	46 (100)	—
R1	0 (0)	1 (1.7)		0 (0)	
Postoperative complications, *n* (%) Clavien–Dindo ≥ grade II					
Yes	8 (40.0)	20 (35.7)	0.73	13 (28.2)	0.35
No	12 (60.0)	36 (64.2)		33 (71.7)	
pT category, *n* (%)^a^					
0	4 (20.0)	5 (8.9)	0.092	0 (0)	0.003
1	2 (10.0)	6 (10.7)		1 (2.1)	
2	0 (0)	4 (7.1)		7 (15.2)	
3	6 (30.0)	29 (51.7)		12 (26.0)	
4a	8 (40.0)	10 (17.8)		25 (54.3)	
4b	0 (0)	2 (3.5)		1 (2.1)	
pN category, *n* (%)^a^					
0	6 (30.0)	17 (30.3)	0.70	6 (13.0)	0.40
1	3 (15.0)	14 (25.0)		6 (13.0)	
2	6 (30.0)	11 (19.6)		17 (36.9)	
3	5 (25.0)	14 (25.0)		17 (36.9)	
pM category, *n* (%)^a^					
0	20 (100)	55 (98.2)	0.43	46 (100)	—
1	0 (0)	1 (1.7)		0 (0)	
pStage, *n* (%)^a^					
0	4 (20.0)	5 (8.9)	0.24	0 (0)	0.004
I	1 (5.0)	8 (14.2)		0 (0)	
II	3 (15.0)	17 (30.3)		13 (28.2)	
III	12 (60.0)	25 (44.6)		33 (71.7)	
IV	0 (0)	1 (1.7)		0 (0)	
Tumor regression grade, *n* (%)					
0	1 (5.0)	1 (1.7)	0.50		
1	12 (60.0)	42 (75.0)			
2	3 (15.0)	8 (14.2)			
3	4 (20.0)	5 (8.9)			
Adjuvant chemotherapy, *n* (%)					
Yes	11 (55.0)	48 (85.7)	0.006	12 (26.0)	0.025
No	9 (45.0)	8 (14.2)		34 (73.9)	

^a^ In the NAC‐Older and NAC‐Young groups, “pT,” “pN,” “pM,” and “pStage” refer to “ypT,” “ypN,” “ypM,” and “ypStage,” respectively.

#### 3.1.4 Survival

**FIGURE 2:**
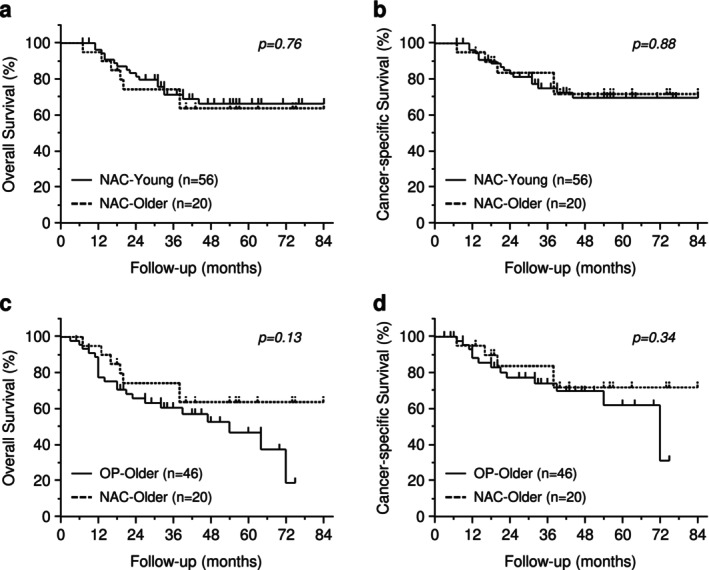
Kaplan–Meier curves for survival comparing NAC‐Young and NAC‐Older groups, or OP‐Older and NAC‐Older groups. Overall survival (a) and cancer‐specific survival (b) between NAC‐Young (*n* = 56) and NAC‐Older groups (*n* = 20). Overall survival (c) and cancer‐specific survival (d) between OP‐Older (*n* = 46) and NAC‐Older groups (*n* = 20).

Figure 2a,b shows Kaplan–Meier estimates of OS and CSS in NAC‐Older and NAC‐Young groups. The median follow‐up time was 41.5 months (7.0–84.0 months). OS and CSS did not significantly differ between the two groups. Similarly, after stratification by clinical stages, no significant difference in OS and CSS was detected in cStage II and III between the two groups (Figure S1).

### 3.2 Comparison Between NAC‐Older and OP‐Older Groups (Cohort 2)

#### 3.2.1 Patient Characteristics

Subsequently, we investigated the treatment results between the NAC‐Older and OP‐Older groups. Table 1 summarizes the patient background characteristics. There were 20 and 46 patients in the NAC‐Older and OP‐Older groups, respectively. The median age of the NAC‐Older group was younger than the OP‐Older group (*p* < 0.001). There were no significant differences in sex, ECOG‐PS, ASA‐PS, BMI, histological type, and cStages, but two patients in the OP‐Older group were ECOG‐PS 2–3. The NAC‐Older group had tumors located in esophagogastric junction more frequently.

#### 3.2.2 Perioperative and Pathological Findings

Table 3 summarizes the perioperative and pathological findings. Subtotal esophagectomy and total gastrectomy were more likely to be performed in the NAC‐Older group (*p* < 0.001), and accordingly, blood loss was significantly more in the NAC‐Older group (*p* = 0.001). R0 resection was achieved in 100% of patients in both groups, and the incidence of postoperative complications was not different between the two groups (*p* = 0.35). The NAC‐Older group tended to have lower pT and pStage diseases than the OP‐Older group, which may imply the downstaging after NAC. Adjuvant chemotherapy was administered less frequently in the OP‐Older group (*p* = 0.025).

#### 3.2.3 Survival

**FIGURE 3:**
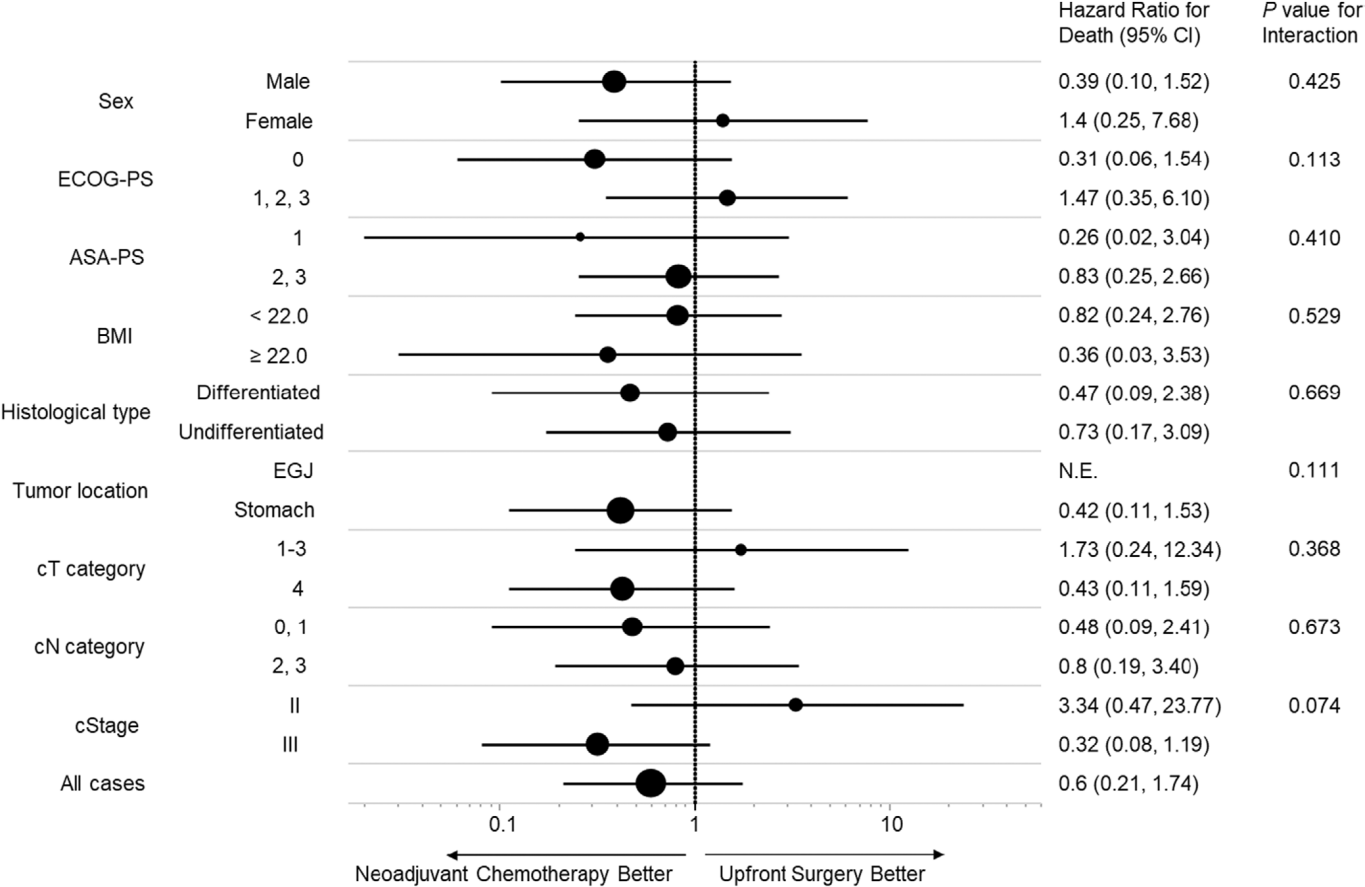
Forest plot of hazard ratios and 95% confidence intervals (CIs) for cancer‐specific survival in 66 older patients with gastric cancer stratified by baseline characteristics: Sex, ECOG‐PS, ASA‐PS, BMI, histological type, tumor location, cT category, cN category, and cStage.

**FIGURE 4:**
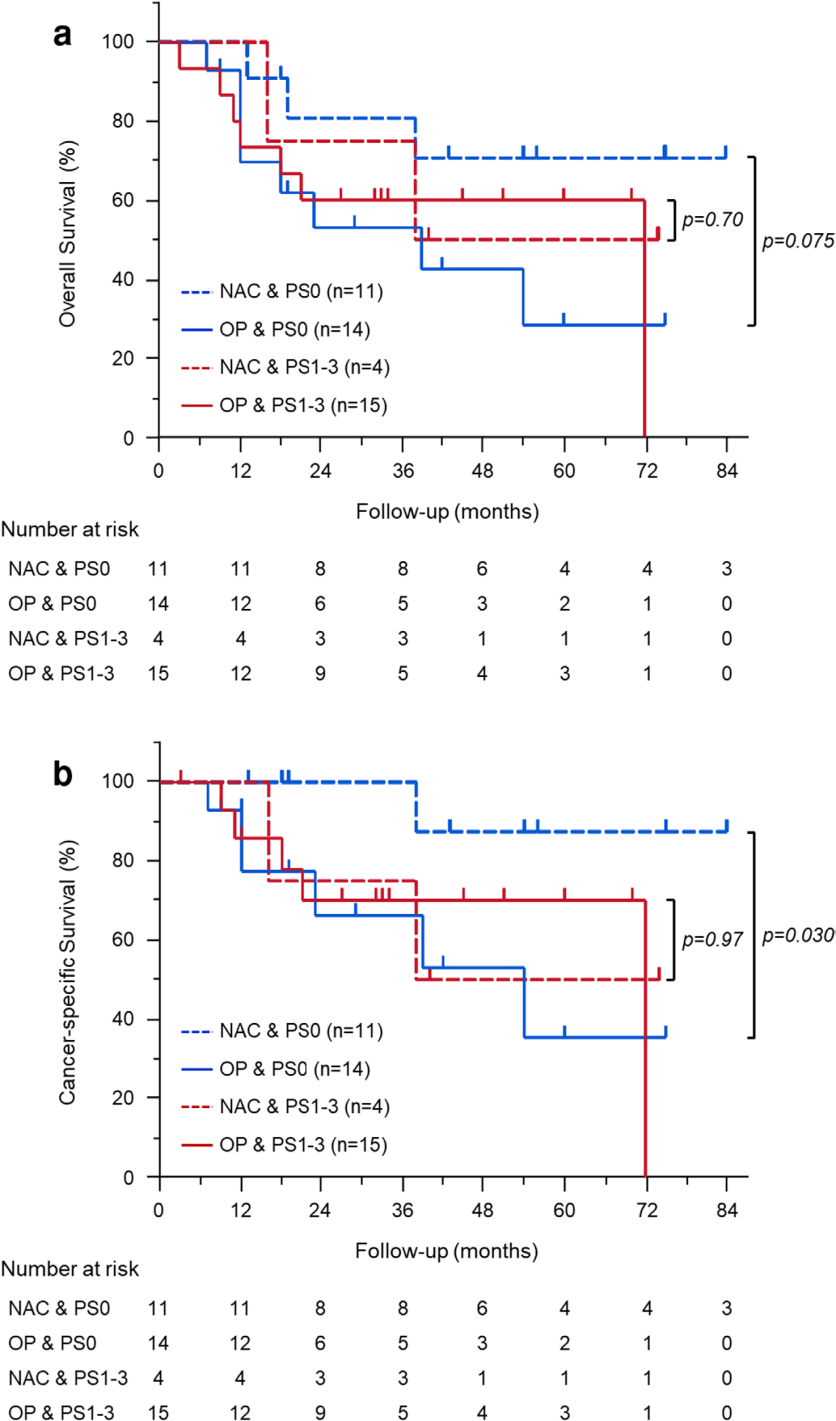
Kaplan–Meier curves for survival in cStage III patients with gastric cancer (*n* = 44). Overall survival (a) and cancer‐specific survival (b) comparing groups stratified by the administration of neoadjuvant chemotherapy and performance status.

Figure 2c,d shows Kaplan–Meier OS and CSS estimates in NAC‐Older and OP‐Older groups. The median follow‐up time was 34.0 months (3.0–84.0 months). There was no significant difference in OS and CSS between the two groups. After stratification by clinical stages, both OS (*p* = 0.097) and CSS (*p* = 0.074) tended to be better in the NAC‐Older than the OP‐Older groups in cStage III (Figure 2c,d). In contrast, there was no significant difference in OS and CSS in cStage II (Figure 2a,b). In the subgroup analysis for CSS, the NAC‐Older group had a better prognosis in patients with ECOG‐PS 0 and cStage III, with a relatively small *p*‐value for interaction in ECOG‐PS and cStage (Figure 3). Consequently, we conducted additional survival analysis between NAC‐Older and OP‐Older groups, accounting for ECOG‐PS (0 vs. 1–3) in cStage III. The results revealed that CSS (*p* = 0.030) was significantly better in NAC‐Older patients compared to OP‐Older patients with ECOG‐PS 0 and cStage III, along with a trend toward improved OS (*p* = 0.075). In contrast, no significant differences in OS and CSS were observed between NAC‐Older and OP‐Older patients with ECOG‐PS 1–3 and cStage III (Figure 4).

## 4 Discussion

In this study, the safety and efficacy of preoperative chemotherapy using DOS or SOX regimen were investigated in older patients with resectable advanced GC. The results demonstrated that preoperative chemotherapy was well tolerated and could be safely administered even in older patients, if the regimen and dosage are adjusted. The older group received a less toxic SOX regimen and reduced initial doses compared to the young group. However, there was no difference in the incidence of adverse events associated with NAC, R0 resection rate, or occurrence of postoperative complications. Regarding treatment efficacy, no significant differences in CSS and OS were observed between NAC‐Older and NAC‐Young groups or between NAC‐Older and OP‐Older groups. Nevertheless, after stratification by ECOG‐PS and cStages, the NAC‐Older group with cStage III and ECOG‐PS 0 showed better CSS and OS compared to the OP‐Older group with cStage III and ECOG‐PS 0, suggesting a potential clinical benefit in the specific subset.

In Asia, upfront surgery plus adjuvant chemotherapy is the standard therapy, as demonstrated by the ACTS‐GC and CLASSIC trails [16, 17]. However, because these trials only enrolled patients aged ≤ 80 years, the efficacy of adjuvant chemotherapy in older patients remains unclear, although some small‐scale studies have suggested its potential efficacy in this population [18, 19]. To address this gap, JCOG1507 is currently underway to evaluate the significance of adjuvant chemotherapy in older patients aged ≥ 80 years [20]. Additionally, NAC has been actively developed in Asia, with key studies including the PRODIGY, RESOLVE, JCOG0501, and JCOG1704 trials, all of which targeted patients aged ≤ 75 years [7, 8, 9, 11, 21]. However, evidence for the efficacy of NAC in older patients remains lacking. Since older patients are more likely to be affected by surgical damage, which may impair their ability to start and complete adjuvant chemotherapy owing to the low tolerance of older patients [22], preoperative chemotherapy may be beneficial for this population. Therefore, this study was designed to investigate the feasibility and efficacy of NAC in older patients aged ≥ 75 years. Indeed, a retrospective cohort study of esophageal cancer indicated that NAC was more effective in older patients with ECOG‐PS 0 than those with ECOG‐PS 1–2 [23]. Similarly, in this study, older patients with cStage III and ECOG‐PS 0 in the NAC group demonstrated better prognoses than those in the OP‐Older group, and in this cohort, patients with ECOG‐PS 0 compared to those with ECOG‐PS 1–3 were more likely to complete three courses of NAC (54.5% vs. 25.0%) and receive adjuvant chemotherapy (72.7% vs. 50.0%). This suggests that even in older patients with advanced GC of cStage III, perioperative treatment with NAC may provide clinical benefit if their performance status is good. These findings should be validated in larger cohorts in the future.

DOS therapy is a high‐response regimen; however, it is associated with a relatively high incidence of adverse events compared to SOX therapy. The incidence of grade 3 or higher adverse events was higher with DOS therapy, including neutropenia (DOS, 79.6%; SOX, 8.3%), febrile neutropenia (35.9%, 8.3%, respectively), and anorexia (29.6%, 8.3%, respectively) [8, 9, 11]. One of the reasons for the higher incidence of hematological adverse events with DOS therapy may be the increased sensitivity of Asian people to docetaxel [24]. Furthermore, previous clinical trials of DOS therapy in GC primarily enrolled younger patients, resulting in a lack of safety data for older patients [7, 11, 25]. To address this question, we investigated the safety profile of NAC in older patients. Accordingly, this study observed no significant differences in overall toxicity between NAC‐Older and NAC‐Young groups. This finding might be attributed to the older patients receiving the less toxic SOX regimen and a reduced initial dose of chemotherapy compared to younger patients. Furthermore, owing to the reduced initial dose of chemotherapy, the proportion of patients requiring mid‐course dose reductions was comparable between the young and older patients. These findings suggest that the regimen and dose of NAC were appropriately adjusted based on the patient's condition, including age and comorbidities, especially in older patients.

Regarding treatment efficacy, previous studies have demonstrated that the DOS regimen achieves a higher response rate than the SOX regimen [25]. However, in this study, despite the frequent use of the SOX regimen and reduced initial dose of chemotherapy in the older patients, the NAC‐Older group achieved a pCR rate of 20.0% and a major pathological response rate of 35.0%. These results were consistent with those of JCOG1704, which reported a pCR rate of 24% and a major pathological response rate of 57% [11]. This outcome may reflect the benefit of administering an appropriate NAC regimen and initial dose for older patients. In other words, considering the feasibility and efficacy of NAC observed in this study, the SOX regimen or a reduced initial dose of chemotherapy may be a more suitable NAC regimen for older patients. Further investigations are warranted to determine the optimal NAC regimen for this population.

This study had several limitations. It was a retrospective study conducted on a small number of patients at a single institution, and there were clear differences in baseline characteristics between the groups. The NAC‐Older group tended to have a higher prevalence of EGC and better overall physical condition, making them more likely to receive adjuvant chemotherapy compared to the OP‐Older group. Furthermore, in this study, the number of NAC courses administered and the proportion of patients receiving adjuvant chemotherapy were lower in the NAC‐Older group compared to the NAC‐Young group, likely due to differences in their overall physical condition. Nevertheless, no significant difference in prognosis was observed between the two groups. This may be partly attributed to the high pathological response rate observed in NAC‐Older group. However, since the impact of potential confounding factors may have affected the outcomes, a larger prospective study will be necessary to validate these findings in patients with similar characteristics.

In conclusion, NAC with S‐1‐based chemotherapy was safely administered even in older patients with locally advanced GC by adjusting the regimen or chemotherapy dose. In older patients with cStage III disease and good performance status, NAC administration was associated with a favorable prognosis. These findings suggest that NAC with DOS or SOX regimens could be a feasible and effective treatment option for older patients with GC with advanced‐stage disease and good overall condition.

## Author Contributions

**Kota Kawabata:** writing – original draft, conceptualization, investigation, validation, writing – review and editing, project administration. **Takuro Saito:** writing – review and editing, supervision, conceptualization, project administration. **Yukinori Kurokawa:** supervision, conceptualization. **Kazuyoshi Yamamoto:** supervision. **Tsuyoshi Takahashi:** supervision. **Koji Tanaka:** supervision. **Tomoki Makino:** supervision. **Kiyokazu Nakajima:** supervision. **Hidetoshi Eguchi:** supervision. **Yuichiro Doki:** supervision.

## Ethics Statement

All procedures followed were in accordance with the ethical standards of the responsible committee on human experimentation (institutional and national) and the Helsinki Declaration of 1964 and later versions. The Human Ethics Review Committee of Osaka University Graduate School of Medicine approved the protocol for this retrospective study (Approval ID: 21440). Each participant provided written informed consent for study participation.

## Conflicts of Interest

All authors report no conflicts of interest or financial ties with any of the firms mentioned in this report. Y. Kurokawa is an Associate Editor of Annals of Gastroenterological Surgery. Y. Doki is an Editorial Board member of Annals of Gastroenterological Surgery.

## Supporting information


Figure S1.
Figure S2.

## Data Availability

The authors will make study raw data available to other researchers upon request from the corresponding author.
