# Supplementary figures and images for: Significance of Neoadjuvant S‐1‐Based Chemotherapy for Older Patients With Locally Advanced Gastric Cancer
Source: Ann Gastroenterol Surg. 2025 May 31;9(6):1163–73. doi: 10.1002/ags3.70049 (PMC12586952; doi:10.1002/ags3.70049)

supplementary figure1

**a**

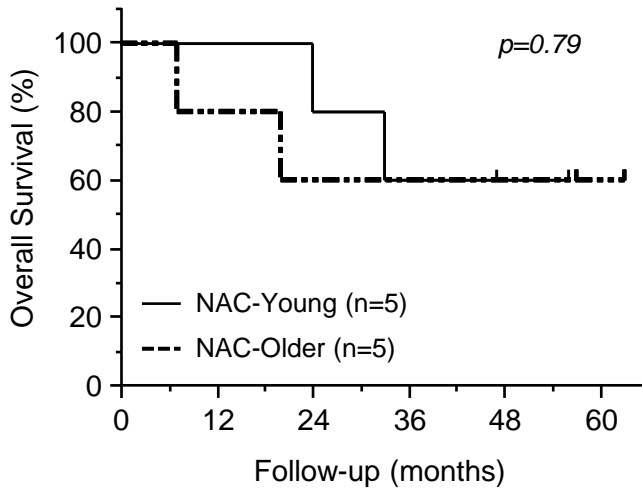

**b**

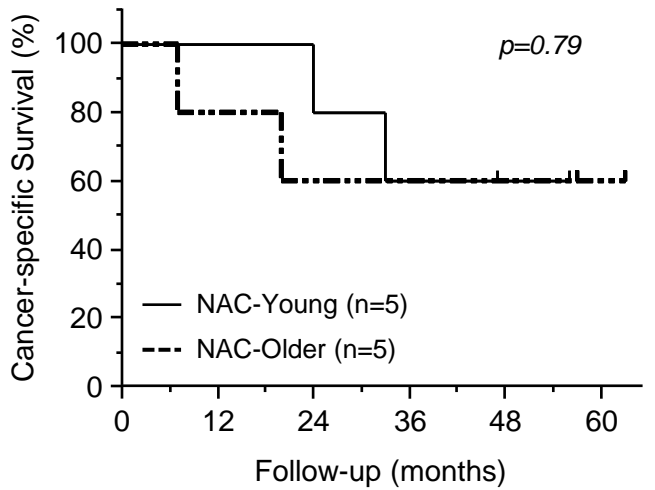

**c**

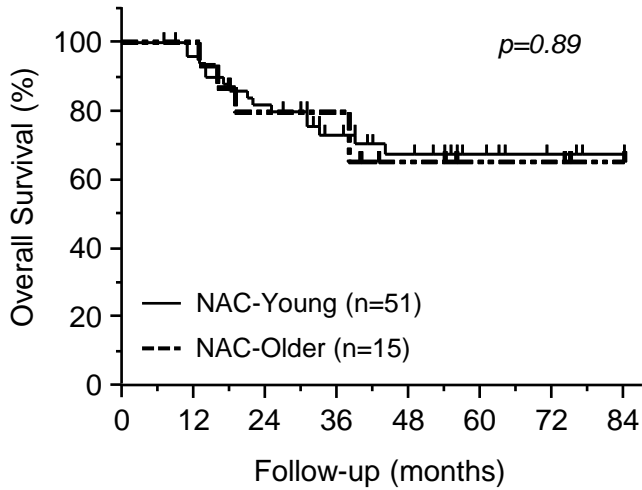

**d**

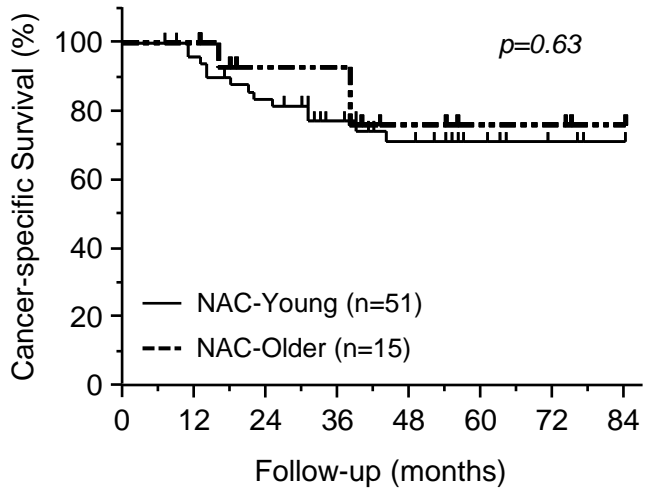

supplementary figure2

**a**

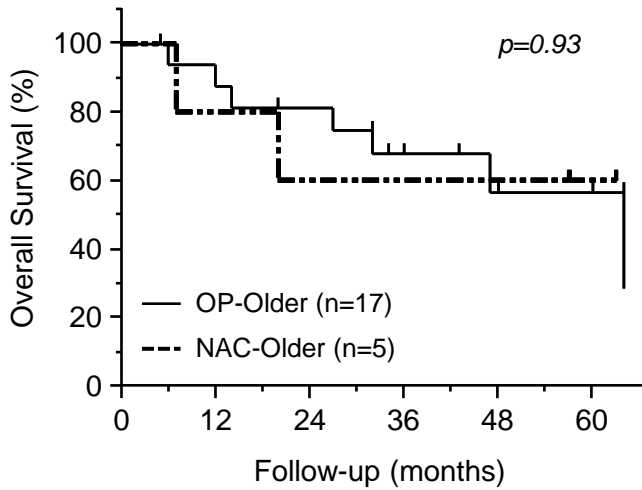

**b**

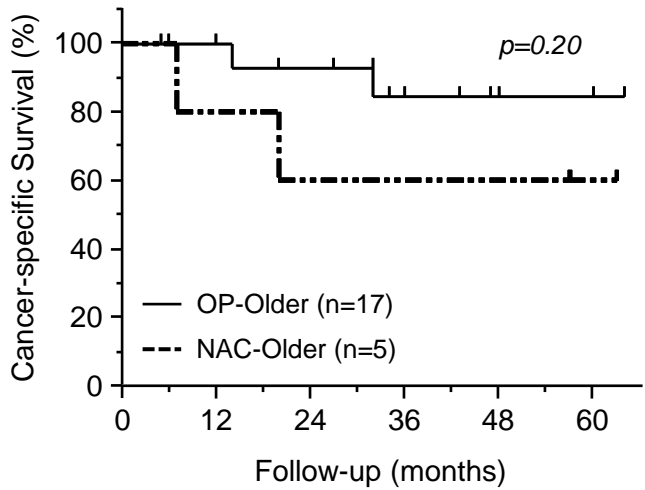

**c**

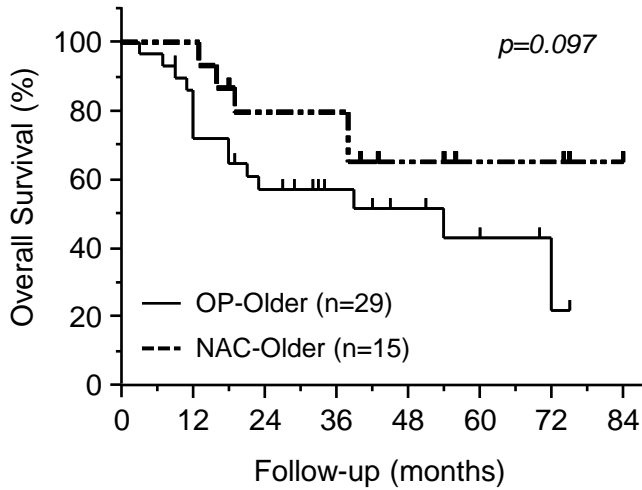

**d**

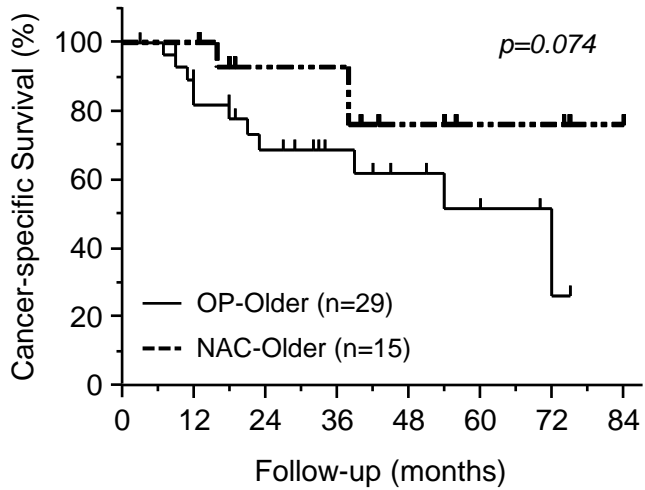

Supplement: Supplementary file 1 — Figure S1. Figure S2. [file AGS3-9-1163-s001.pdf]
